# Generative AI in structure-based drug discovery

**DOI:** 10.1186/s12915-026-02575-x

**Published:** 2026-03-21

**Authors:** Zhuoya Zhong, Jacob D. Durrant

**Affiliations:** Department of Biological Sciences, University of Pittsburgh, Pittsburgh, PA USA

**Keywords:** Generative artificial intelligence, Computer-aided drug design, Structure-based drug design, De novo molecular generation, Lead optimization, Deep learning

## Abstract

Generative artificial intelligence is reshaping how researchers discover protein-binding compounds and develop them into drug candidates. Unlike traditional methods that screen existing molecules, structure-based generative AI designs novel compounds tailored to a protein’s three-dimensional binding pocket. This review outlines how these approaches are applied in early drug discovery, focusing on general principles. We categorize methods according to their generative modeling paradigms and their strategies for using structural data to guide molecular design, distinguishing de novo incremental builders from models that generate full structures. We also survey lead-optimization techniques, highlighting a recent shift toward generation-driven medicinal chemistry.

## From hits to leads: the rising role of generative AI in drug design

The overall aim of drug discovery is to develop therapies that improve health by modulating the activity of biomolecules and cells. Small molecules (i.e., low-molecular-weight organic compounds) are particularly useful as drugs because they can be designed for oral delivery and can be chemically modified via established synthetic routes to improve activity. Such molecules can modulate diverse biomolecular targets, including proteins, nucleic acids, lipid bilayers, and even metal cations. Among these, proteins are especially attractive because their complex, three-dimensional structures frequently form well-defined binding pockets lined with amino acids that can form specific, complementary interactions with small-molecule drugs.

Identifying a small molecule “hit” that binds to a protein target is a crucial first step in many drug-discovery campaigns. Hits typically have modest affinity and may lack suitable absorption, distribution, metabolism, excretion, or toxicity (ADMET) profiles. In the subsequent lead-optimization phase, researchers transform hits into more promising “lead” compounds by chemically modifying them to overcome such challenges.

Machine learning has long been foundational in both hit identification and lead optimization. Historically, most models have been trained to assess (or score) existing molecules, helping researchers sift through large databases to find compounds most likely to bind a target or exhibit favorable properties. In virtual screening, for example, these models evaluate thousands or millions of known compounds, ranking them by predicted affinity to identify candidates worth testing experimentally.

The recent rise of generative AI, which creates novel molecular structures rather than selecting from existing ones, represents an important new frontier in computer-aided drug design. This review focuses on the transformative impact of generative AI applied to both de novo hit generation and lead optimization. We survey methods published primarily within the last 5 years, with a focus on target-aware methods that leverage the 3D geometry and chemical environment of the protein binding site to guide molecular generation.

## Structure-based de novo hit generation: creating molecules from scratch

The goal of structure-based de novo design is to create entirely new hit compounds. In this section, we organize recent generative AI approaches by their molecular generation strategy (Table 1), highlighting specific tools and how each leverages the structure of the protein binding site.

**Table 1:** Overview of select generative AI tools for de novo structure-based drug discovery

Tool	Inc. Build	At Once	Paradigm	Architecture
3D-Generative-SBDD [1]	X		Autoregressive	GNN
GraphBP [2]	X		Autoregressive	GNN
Pocket2Mol [3]	X		Autoregressive	GNN
DeepLigBuilder [4]	X		Autoregressive	GNN
Lingo3DMol [5]	X		Autoregressive	Transformer
TargetVAE [6]	X		VAE	Hybrid
AutoGrow4 [7]	X		Evolutionary	
JANUS [8]	X		Evolutionary	
SMILES-RNN [9]	X		RL	RNN
TargetDiff [10]		X	Diffusion	GNN
DiffSBDD [11]		X	Diffusion	GNN
DiffBP [12]		X	Diffusion	GNN
PMDM [13]		X	Diffusion	GNN
PoLiGenX [14]		X	Diffusion	GNN
PILOT [15]		X	Diffusion	GNN
DRUGFLOW [16]		X	Diffusion	GNN
LiGAN [17]		X	VAE	CNN

De novo generative AI methods are organized by molecular generation strategy. “Inc. Build” and “At Once” indicate that the method builds molecules incrementally or all at once, respectively. Abbreviations: *CNN*, convolutional neural network; *GNN*, graph neural network; *RL*, reinforcement learning; *RNN*, recurrent neural network; *VAE*, variational autoencoder. “Hybrid” indicates a combination of multiple architectures (e.g., RNN, Transformer, and GNN in the case of TargetVAE)

### Building small molecules incrementally

Many de novo methods build molecules sequentially, one atom or fragment at a time. This stepwise, structure-aware process allows the model to make context-dependent decisions at each step, taking into consideration both the protein’s binding pocket and the partially constructed molecule.

#### Autoregressive approaches: sequential construction

Autoregressive models decompose the complex task of generating an entire molecule into a series of simpler predictions. At each step, these models make two interconnected decisions: (1) which atom type or molecular fragment to add next, and (2) where to add that atom or fragment.

Many autoregressive approaches use graph neural networks (GNNs) to make these stepwise decisions. GNNs represent small molecules and proteins as graphs where atoms are nodes and chemical bonds are edges. By passing information between connected nodes, GNNs learn to recognize patterns in molecular connectivity and the three-dimensional positioning of atoms. For example, 3D-Generative-SBDD [1] learns to predict the likelihood of finding a particular atom type at any 3D position within the binding pocket. At each step, it selects an atom to place based on these learned probabilities, with each new atom updating the molecular context for subsequent predictions. GraphBP [2] similarly builds molecules atom-by-atom but introduces a local coordinate system, making it robust to different protein orientations. Finally, Pocket2Mol [3] jointly predicts the position and type of the next atom as well as the chemical bond connecting it to the existing structure. This integrated approach helps prevent unrealistic structures, a common challenge for sequential generation methods.

To optimize for desirable properties more explicitly, some autoregressive GNN approaches combine the generative model with an external, goal-oriented search algorithm. A prominent example is DeepLigBuilder [4], which uses a structure-based scoring function to assess the growing molecule, prioritizing paths that lead to compounds with high predicted binding affinities.

Unlike these autoregressive GNN methods, Lingo3DMol [5] uses an autoregressive transformer model. Originally designed for language tasks, transformers excel at processing sequential data through their “attention mechanism,” which dynamically weighs the relevance of preceding elements when making predictions. Lingo3DMol applies this principle to molecular design, generating molecules fragment-by-fragment just as a language model writes sentences word-by-word. The model first identifies key interaction points within the protein’s binding pocket, then selects fragments sequentially while simultaneously predicting chemical connectivity and 3D placement.

#### Sequential generation via VAEs

Variational autoencoders (VAEs) can also be used to build molecules sequentially. These generative models consist of two components: an encoder that learns to compress input data (e.g., molecular structures) into more abstract representations, and a decoder that learns to reconstruct the original data from those compressed representations. The VAE organizes the compressed representations into a “latent space” such that similar inputs map to adjacent latent-space regions. By decoding representations sampled from this compressed space, a VAE decoder can generate new data that resembles the training examples.

TargetVAE [6] is a good example of this approach. It uses two separate recurrent neural networks (RNNs) to learn how to encode and decode (reconstruct) text-based representations of small molecules. In brief, RNNs process information serially, maintaining a form of memory as they move through the data one element at a time. Because this progressive mechanism allows earlier elements to influence the interpretation of later ones, RNNs are naturally suited to generating sequential data such as text and text-based molecular representations.

Once training is complete, the TargetVAE RNN decoder can be used to build text-based molecular representations incrementally, symbol by symbol, given abstract representations chosen from the latent space. The key challenge is determining which region of the latent space to sample from. A separate model called the Protein Multimodal Network (PMN) addresses this challenge by encoding a protein’s sequence and 3D structure into a single representation. TargetVAE then uses that protein representation to guide sampling toward regions of the latent space likely to yield molecules well-suited to the given protein. Notably, users need not indicate the location of any binding pocket because the PMN considers the protein’s complete sequence and structure. Because of this design choice, TargetVAE is particularly well-positioned for exploring binding at cryptic or allosteric sites that might be overlooked by pocket-dependent methods.

#### Evolution in silico: genetic algorithms for hit discovery

Like autoregressive methods, evolutionary algorithms also build molecules incrementally, but they are guided by principles learned from natural selection. These algorithms begin with a population of very small chemical fragments and iteratively apply chemical modifications to mimic mutation (adding, removing, or modifying molecular substructures) and crossover (combining parts from two parent molecules). Crucially, these genetic operators implement molecular changes without regard for the protein’s 3D geometry. However, the protein structure is essential for guiding selection through a fitness evaluation (often based on structure-aware predicted affinities), which determines which molecules advance to the next generation.

Several recent tools use this evolutionary strategy. For example, AutoGrow4 [7] is an open-source genetic algorithm that starts with a population of small chemical fragments and applies mutation and crossover operations, with molecular docking scores guiding selection. The algorithm iteratively evaluates the compounds of each generation, retaining molecules with favorable binding scores and using them as templates for subsequent rounds of modification. Similarly, the JANUS framework [8] uses a dual-population approach to balance exploration and exploitation. One population broadly searches chemical space while the other refines high-fitness regions, with members exchanged between the two. Unlike AutoGrow4, JANUS requires users to implement their own fitness functions. However, as the authors demonstrate, docking can be used effectively to judge the fitness of the evolving compounds. In both AutoGrow4 and JANUS, successive generations are progressively enriched with molecules that have improved predicted properties, as weaker candidates are eliminated and successful ones propagate forward.

#### Trial-and-error design: reinforcement learning for de novo generation

Reinforcement learning offers another strategy for training models to construct new molecules incrementally. In this framework, a computational “agent” improves its decision making by interacting with an environment. The agent performs an action, and the environment provides feedback as a “reward” or “penalty.” The agent’s goal is to learn a strategy that maximizes cumulative reward over time.

SMILES-RNN [9] is one example of a de novo molecular generation method that adopts this approach. It uses RNNs to construct text-based molecular representations sequentially, symbol by symbol. The RNN is first pre-trained on a large dataset of known drug-like molecules to learn valid chemical syntax and structural patterns. Reinforcement learning then further trains this model, rewarding it for generating molecules with desirable properties. By incorporating protein-aware docking scores into the reward function, the authors successfully designed adenosine A₂A receptor ligands that were subsequently validated experimentally.

### Building small molecules all at once

Some de novo methods generate entire molecular structures simultaneously rather than building them incrementally. These approaches consider the binding pocket as a whole and produce complete compounds in a single generative process.

#### From noise to molecules: generative diffusion models

Generative diffusion models learn to iteratively transform random noise into coherent, structured output through a gradual denoising process, typically by using GNNs to learn the denoising step. Many generate molecules conditioned on the protein binding pocket alone. Notable examples include TargetDiff [10] and DiffSBDD [11], which predict both small-molecule atomic coordinates and atom types; DiffBP [12], which additionally uses an initial prediction of the molecule’s size and centre of mass to guide generation; and PMDM [13], which accounts for interactions such as chemical bonds and van der Waals forces during generation.

In contrast, some diffusion methods use additional factors beyond the binding pocket to guide the diffusion process. For example, PoLiGenX [14] can optionally incorporate a seed molecule to guide generation. When users disable the seed molecule’s influence, PoLiGenX functions as a pure de novo generator, creating compounds conditioned only on the binding pocket. When enabled, PoLiGenX uses the seed to guide the denoising process, producing new molecules that maintain the seed’s overall shape while exploring different chemical structures optimized for the target. A tuneable parameter controls the seed’s influence, allowing users to balance pure de novo generation against reference-guided exploration.

Still other diffusion approaches actively steer molecular generation toward compounds with desirable properties. For example, PILOT [15] evaluates partially formed molecules during generation for properties such as synthetic accessibility and affinity. In contrast, DRUGFLOW [16] steers generation using “preference alignment”. The model is first pre-trained on protein/small-molecule binding data to learn how to generate drug-like molecules that fit into a given binding pocket. It is then further refined by training on paired examples of molecules, where each pair consists of one molecule with favorable properties (e.g., molecular docking scores) and another with unfavorable properties. Through this additional training phase, the model learns to prefer molecules that resemble the favorable examples.

#### One-shot generation via VAEs

Just as VAEs can generate molecules incrementally (see above), they can also be trained to generate complete molecules in a single step. VAEs based on convolutional neural networks (CNNs) are well suited to this task. In brief, CNNs excel at processing grid-like data by learning to recognize spatial patterns, from simple features to complex structures. In drug discovery applications, a protein/small-molecule complex is first converted into a 3D grid of voxels (essentially 3D pixels), where each voxel contains information about nearby atoms. The CNN then learns to extract important spatial and chemical patterns from these molecular grids.

LiGAN [17] uses CNNs to simultaneously learn how to (1) encode protein/small-molecule voxel grids into abstract representations and (2) decode (reconstruct) the small-molecule grids from those same representations. To generate new molecules, LiGAN samples from the range of encoded representations, decoding new voxel grids that show where small-molecule atoms should be located. When provided with information about a specific protein pocket, the decoder generates small-molecule grids that fit that pocket’s shape and chemistry. These grids are then converted into actual molecular structures through post-processing algorithms that position atoms and determine chemical bonds based on interatomic distances.

## Structure-based lead optimization: refining known binders

Structure-based lead optimization involves chemically modifying existing early-stage hits to overcome poor ADMET properties or binding affinities. Below, we organize recent generative AI approaches by their lead-optimization strategy (Table 2), highlighting specific tools and how they incorporate protein structure data to guide optimization.

**Table 2:** Overview of select generative AI tools for structure-based lead optimization

Tool	Add	Link	Paradigm	Architecture
PMDM [13]	X	X	Diffusion	GNN
DiffSBDD [11]	X	X	Diffusion	GNN
DiffBP [12]	X		Diffusion	GNN
AutoGrow4 [7]	X		Evolutionary	
JANUS [8]	X		Evolutionary	
DeepLigBuilder [4]	X		Autoregressive	GNN
DeepFrag [18]	X		Fragment selection	CNN
FRAME [19]	X		Autoregressive	GNN
DiffDec [20]	X		Diffusion	GNN
LibINVENT [21]	X		RL	RNN
3D-Generative-SBDD [1]		X	Autoregressive	GNN
DiffLinker [22]		X	Diffusion	GNN
Link-INVENT [23]		X	RL	RNN

Generative AI methods for lead optimization are organized by lead-optimization strategy. “Add” and “Link” indicate that the method performs lead optimization via fragment addition or fragment linking, respectively. Abbreviations: *CNN*, convolutional neural network; *GNN*, graph neural network; *RL*, reinforcement learning; *RNN*, recurrent neural network

### Step by step: lead optimization via fragment addition

Fragment addition methods modify an existing small molecule by attaching new chemical groups to the core scaffold, sometimes after first deleting an existing group at the same position (i.e., fragment swapping). Structure-based generative AI tools guide these additions using the protein binding site to identify and position chemically appropriate fragments.

#### Multi-functional de novo and lead optimization approaches

Many tools for de novo design are also useful for lead optimization via fragment addition. For example, diffusion tools such as PMDM [13], DiffSBDD [11], and DiffBP [12] also allow users to generate decorating moieties around a fixed scaffold. Evolutionary algorithms such as AutoGrow4 [7] and JANUS [8] can also be readily adapted for lead optimization by starting with a known hit as the initial seed, rather than building from a small molecular fragment. Similarly, the autoregressive/GNN tool DeepLigBuilder can start from a known small-molecule binder [4].

#### Fragment selection using CNNs

Still other tools are specifically designed for fragment-addition lead optimization. For example, DeepFrag [18] uses CNNs to process voxel grids representing both the protein binding pocket and an existing small-molecule binder. Rather than generate atomic coordinates and atom types directly, DeepFrag predicts a molecular “fingerprint” that captures the characteristics of a fragment expected to complement both the existing small-molecule scaffold and the protein binding pocket. This predicted fingerprint is then matched against a library of pre-computed fingerprints, each representing a known molecular fragment, to identify suitable candidates. Importantly, the library can be customized, allowing researchers to restrict selections to synthetically accessible fragments or to explore broader chemical space.

#### Informed sequential expansion: autoregressive GNN methods

FRAME [19] is an example of an autoregressive GNN-based model designed specifically for lead optimization. It breaks down the process of growing a small molecule into individual steps, each step guided by a trained GNN. Given a starting small-molecule binder, FRAME first identifies where on the molecule to add chemical groups, then selects which groups to add based on patterns learned from existing high-affinity, drug-like small molecules.

#### Noise-to-decoration: diffusion for scaffold modification

Unlike the multi-functional diffusion methods described above, DiffDec [20] is a graph-based (GNN) diffusion model designed solely for lead optimization. It holds a molecule’s core constant while generating geometrically and chemically favourable decorating chemical groups well-suited to the binding site. A notable feature is its “fake atom mechanism,” which allows the model to generate new chemical groups of various sizes in a single, integrated process, rather than requiring a separate step to first predict the size. In evaluations, DiffDec successfully regenerated missing chemical groups removed from known binders and separately identified novel chemical additions that improved predicted affinity.

#### Trial-and-error optimization: reinforcement learning

The RNN-based reinforcement-learning framework described above in the context of de novo generation (e.g., SMILES-RNN [9]) can also be adapted for lead optimization. Specifically, the LibINVENT approach [21] also involves pre-training an RNN on drug-like molecules to learn valid chemical syntax and structural patterns. This “agent” is further trained via reinforcement learning to decorate a user-provided molecular core with novel chemical groups. The agent itself operates only on the small molecule, but with minimal coding, users can implement a reward function that guides its learning via feedback from the protein structure (e.g., molecular docking scores). In such cases, the 3D geometry of the binding pocket would indirectly steer the generation process, though this capability was not explicitly demonstrated in the original work [21].

### Bridging the gap: lead optimization via fragment linking and scaffold hopping

Fragment linking and scaffold hopping (core replacement) are related lead-optimization strategies that involve connecting chemical fragments through a central linking structure. In fragment linking, the goal is to link separate fragments, each binding different subpockets, into a single molecule with improved binding. Scaffold hopping extends this concept by removing the central scaffold of a known active compound and replacing it with a structurally novel core (linker) that maintains the positions of the decorating substructures. The primary challenge in both strategies is discovering a linker or scaffold that itself complements the protein pocket while preserving the interactions of the existing functional groups.

#### Multipurpose de novo, fragment merging, and linker design models

Two of the diffusion models capable of de novo generation and fragment-addition lead optimization, PMDM [13] and DiffSBDD [11], are also useful for fragment linking. These versatile models treat the existing functional groups as fixed fragments or “seeds,” and the denoising process generates the necessary atoms to bridge the gap via a novel linker or core. Essentially, they “in-paint” the missing chemical matter by iteratively refining random noise until a chemically and geometrically plausible linker emerges, respecting the 3D spatial constraints of the protein’s binding pocket.

Some GNN-based models are also multipurpose. For example, 3D-Generative-SBDD [1], described earlier in the context of de novo design, can be adapted for linker design by providing two fixed fragments as initial input. The atom-by-atom generation process proceeds to fill the space between the fragments, growing a linker guided by the geometry of both the fragments and binding site.

#### Models designed specifically for fragment linking

In contrast to these multi-purpose models, some models are specifically trained for fragment linking. For example, DiffLinker [22] is a diffusion model that operates in two stages. First, a GNN predicts possible linker sizes based on the fragments’ positions and orientations. Then, a graph-based (GNN) diffusion model generates a linker by refining noise into a valid chemical structure that connects the fragments, optionally taking into consideration the predicted size distribution. DiffLinker is notable in its ability to connect an arbitrary number of fragments simultaneously, whereas many methods link only a single pair.

Reinforcement learning provides another strategy for linker design. Link-INVENT [23] adopts an approach similar to LibINVENT, described above, in that it uses RNNs to sequentially construct text-based molecular representations of suitable linkers, symbol-by-symbol. Link-INVENT begins with a pre-trained model that learns valid chemical syntax. Reinforcement learning then optimizes this initial model for the specific task of bridging two user-provided molecular fragments. Although the agent works directly with text-based representations of the small molecule, reward functions incorporating molecular docking scores allow the protein’s 3D structure to indirectly influence the learning process. The binding pocket’s geometry thus indirectly guides generation toward linkers that maintain favorable positioning of the fragments within the target site.

## Challenges and limitations

Despite rapid progress, several critical barriers have slowed the integration of generative AI tools into real-world drug-discovery workflows. Addressing these limitations will be essential for driving widespread adoption.

### The challenge of practical usability and software accessibility

Unfortunately, many generative drug-design tools are challenging to use. They provide only command-line interfaces, and several (e.g., LibINVENT [21], JANUS [8]) can only incorporate protein structural information if the user provides custom computer code. Other tools lack publicly available source code (e.g., DeepLigBuilder [4]) or depend on associated trained weights that are available only upon request (e.g., PoLiGenX [14], PILOT [15]). In terms of usability, Lingo3DMol [5] and DeepFrag [18] are notable exceptions, offering intuitive browser-based interfaces that accept standard PDB files without requiring local installation. The DiffBP manuscript [12] also mentions a web server, though it was unavailable when we attempted to access it in late February 2026.

Even when the software itself is accessible, preparing suitable input data presents its own challenges. Most tools accept standard PDB files as input, but the effort required to prepare protein structures creates additional preprocessing burdens. Aside from repairing missing binding-pocket residues and heavy atoms, users must also navigate inconsistent requirements regarding non-protein components and protonation. For example, many tools ignore non-protein components entirely (e.g., 3D-Generative-SBDD [1], Pocket2Mol [3], TargetVAE [6], DiffSBDD [11], PMDM [13], and DRUGFLOW [16]), while others natively or selectively retain components such as co-factors and structural waters (e.g., Lingo3DMol [5], TargetDiff [10], DeepFrag [18], DiffDec [20], DiffLinker [22]). Protonation handling is similarly inconsistent. Many models were trained on hydrogen-free datasets (e.g., CrossDocked2020 [24]), suggesting users should strip hydrogen atoms prior to input. Yet tools that leverage molecular docking (e.g., AutoGrow4 [7], SMILES-RNN [9], Link-INVENT [23]) typically inherit the protonation requirements of the docking engine. To add to this complication, some tools make protonation optional. Lingo3DMol [5], for example, uses hydrogen atoms (if present) to more accurately determine protein atoms’ hydrogen-bond donor/acceptor status, though it also attempts to determine that status if hydrogen atoms are absent. Finally, we note that some tools also require users to convert their PDB files to method-specific file formats (e.g., GraphBP [2], DiffBP [12], LiGAN [17], FRAME [19]), further adding to the preprocessing burden.

### The challenge of standardized benchmarking and experimental validation

Computational metrics such as predicted binding affinity, synthetic accessibility, and molecular diversity are ultimately only proxies for success; without standardized benchmarking or rigorous wet-lab validation, they cannot definitively confirm whether a generative model produces genuinely viable drug candidates. Unfortunately, most methods reviewed have only been evaluated computationally, and while some publications include head-to-head comparisons (e.g., refs [1–3, 10–15, 20, 22, 23]), the field still lacks a universally applied, cross-method computational benchmark.

Beyond computational benchmarks, even fewer of these tools have been subjected to prospective experimental validation. SMILES-RNN [9], the reinforcement-learning approach for de novo generation, is one notable exception. Thomas et al. [9] used SMILES-RNN to successfully design adenosine A_2_A receptor ligands. Radioligand binding assays confirmed that eight of the nine synthesized compounds were active binders, including three with nanomolar affinity. Similarly, Huang et al. used PMDM [13], the diffusion-based tool for lead optimization, to modify a known CDK2 inhibitor, yielding six novel synthesized derivatives with improved activity, five of which achieved sub-nanomolar potency.

That said, such studies remain exceptions, and the real-world utility of most tools is unproven. Community-driven initiatives such as the CACHE (Critical Assessment of Computational Hit-finding Experiments) challenge [25] are thus vital. Analogous to CASP for protein structure prediction, CACHE provides blind, experimentally validated assessments of computational hit-finding methods generally. Several generative methods have already participated in early CACHE rounds [26–28], and expanding this type of prospective validation will be essential to distinguish which models deliver genuine practical value.

### The challenge of synthesizability and chemical plausibility

Molecular designs that cannot be physically synthesized in a laboratory are functionally useless, regardless of predicted affinity. Unfortunately, in many cases, the gap between computationally generated structures and synthetically accessible, experimentally active compounds remains substantial. For example, several groups have reported [3, 10, 13] that 3D generative methods frequently output strained topologies with excessive three- and four-membered rings (e.g., LiGAN [17], 3D-Generative-SBDD [1], and DiffSBDD [11]) or uncommon seven-membered rings (e.g., TargetDiff [10]), which are difficult to synthesize [15]. Post-processing filters can remove some problematic outputs, but at the cost of reduced throughput and potential bias.

To address this challenge, a few tools incorporate synthetic accessibility directly into the generation phase. For example, AutoGrow4 [7] and LibINVENT [21] can use reaction-based frameworks to inherently constrain outputs to synthetically plausible chemical space. Similarly, the diffusion-based PILOT [15] can actively steer molecular generation by assessing synthetic accessibility scores as molecules take shape during the denoising process. Future work must continue to natively integrate synthetic awareness, whether through reaction-aware generation or scoring functions that penalize intractable designs.

### The challenge of protein flexibility

Proteins are highly dynamic, and binding pockets frequently reshape themselves through side-chain rotations and backbone motions. Treating targets as static, rigid structures severely limits a model’s ability to identify ligands that rely on induced-fit mechanisms or transient cryptic pockets. Unfortunately, nearly all surveyed methods treat the protein as a rigid body. To account for flexibility, users must run separate generative campaigns across multiple independent protein conformations and pool the results.

Among the reviewed tools, only FLEXFLOW, an extension of DRUGFLOW [16], takes a step toward incorporating protein flexibility directly into the generative process. Aside from generating ligand atoms, FLEXFLOW jointly predicts the rotational angles of binding-pocket side chains, allowing them to adopt orientations that complement the generated molecule. While enabling side-chain flexibility represents a meaningful advance, expanding these capabilities to also capture backbone motions and larger-scale conformational changes remains an important frontier.

## Looking ahead: integration and innovation

In summary, generative AI has fundamentally transformed structure-based drug discovery, offering powerful new strategies for identifying novel hits and optimizing existing leads. The field has seen rapid innovation, with a diverse array of methods now capable of tackling key challenges in medicinal chemistry. For de novo design, these tools move beyond traditional virtual screening by actively constructing molecules that complement a protein binding pocket’s specific 3D geometry and chemical features. For lead optimization, they provide a systematic framework for improving small-molecule binding while respecting the spatial constraints imposed by the target protein structure.

As the field continues to mature, we expect generative AI to become increasingly integrated into early-stage drug-discovery workflows, complementing rather than replacing human expertise. Challenges remain, but the rapid pace of innovation suggests that structure-based generative AI will play an increasingly central role in bringing new therapeutics from concept to clinic.

## Data Availability

No datasets were generated or analysed during the current study.
